# Low-molecular-weight heparin in addition to low-dose aspirin for preventing preeclampsia and its complications: A systematic review and meta-analysis

**DOI:** 10.3389/fcvm.2022.1073148

**Published:** 2022-12-09

**Authors:** Li Zheng, Binbin Xia, Yuan Yuan, Yuran Wang, Yan Wang

**Affiliations:** ^1^Department of Pharmacy, China Aerospace Science & Industry Corporation 731 Hospital, Beijing, China; ^2^Department of Pharmacy, Beijing Luhe Hospital Affiliated to Capital Medical University, Beijing, China; ^3^Department of Dermatology, Gansu Provincial Central Hospital, Lanzhou, China; ^4^Obstetrics and Gynecology Department, China Aerospace Science & Industry Corporation 731 Hospital, Beijing, China; ^5^Department of Cardiovascular Medicine, Beijing Hospital, National Center of Gerontology, Institute of Geriatric Medicine, Chinese Academy of Medical Sciences, Beijing, China

**Keywords:** low-dose-aspirin, low-molecular-weight heparin, prevention, preeclampsia, meta-analysis

## Abstract

**Background:**

In this systematic review, we aimed to investigate the efficacy and safety of adding low-molecular-weight heparin (LMWH) or unfractionated heparin to low-dose aspirin (LDA) started ≤16 weeks'gestation in the prevention of preeclampsia (PE) in high-risk women.

**Methods:**

PubMed, Cochrane Library, Embase, and ClinicalTrials.gov databases were searched from their inception to April 2022 for randomized controlled trials (RCTs) that to determine whether the combined treatment of LMWH and LDA is better than single anticoagulant drugs in preventing PE and improving live birth rate of fetus in high-risk women with pregnancy ≤16 weeks. We also searched Embase, OVID MEDLINE and OVID MEDLINE in-process using the OVID platform.

**Results:**

14 RCTs involving 1,966 women were found. The LMWH (or unfractionated heparin) and LDA groups included 1,165 wemen, and the LDA group included 960 women. The meta-analysis showed that the addition of LMWH to LDA reduced the risk of PE (RR: 0.59, 95% CI: 0.44-0.79, *P* < *0.05*), small-for-gestational age (SGA, RR: 0.71, 95% CI: 0.52-0.97, *P* = *0.03*), fetal and neonatal death (RR: 0.45, 95% CI: 0.23-0.88, *P* = *0.02*) and gestational hypertension (RR: 0.47, 95% CI: 0.25-0.90, *P* = *0.02*). It is worth emphasizing that LMWH (or unfractionated heparin) combined with LDA did not increase the risk of bleeding.

**Conclusions:**

LMWH combined with LDA can effectively improve the pregnancy outcome of women with high risk factors for PE and its complications. Although this study showed that combined medication also did not increase the risk of bleeding, but such results lack the support of large sample size studies. The clinical safety analysis of LMWH combined with LDA in patients with PE should be more carried out.

## Introduction

Preeclampsia (PE) is characterized by hypertension and proteinuria, and it is one of the important factors leading to maternal and perinatal death. The incidence rate of all pregnant women is about 2–8% (1, 2). The main manifestations of PE are maternal and multiple organ and system damage, as well as adverse pregnancy outcomes such as fetal growth restriction (FGR) and placental abruption. At the same time, PE can have adverse effects on re-pregnancy. Women with a history of PE are 25–65% likely to have PE again, 3% may have placental abruption, and 10% may have FGR again (3). The causes are multifactorial, including dysinvasion of uterine spiral artery trophoblast, injury of vascular endothelial cells and transitional activation of maternal immune system (4–7). The dysfunction or disorder of hemostasis, coagulation, anticoagulation and fibrinolysis system caused by many factors can eventually lead to the abnormal increase of coagulation function and the decrease of fibrinolysis function, so that the blood is in a hypercoagulable state.

At present, there are evidence-based evidences that the application of aspirin in high-risk groups of PE can effectively prevent the onset of PE and reduce adverse pregnancy outcomes (8, 9). Studies have also confirmed that low molecular weight heparin (LMWH) reduces the incidence of PE, perinatal death and FGR in high-risk pregnant women, and improves the pregnancy outcome of patients (10, 11). In addition, because LMWH cannot pass through the placenta, there is almost no direct risk to the fetus, and massive hemorrhage or placental abruption are rarely observed in pregnant women treated with LMWH (12).

In recent years, some studies have reported the effect and safety of aspirin combined with LMWH in the prevention of PE and its complications. In this study, meta-analysis method was used to conduct a comprehensive evaluation of these related studies to make up for the small sample size of a single study. The primary purpose is to determine whether the combined treatment of LMWH and aspirin is better than single anticoagulant drugs in preventing PE and its complications in high-risk women with pregnancy ≤16 weeks.

## Methods

We performed a meta-analysis and wrote the article by conforming to the requirements illustrated in the Preferred Reporting Items for Systematic Reviews and Meta-Analyses (PRISMA) statement (13). We compared the treatment with LMWH or heparin (with low-dose aspirin) with LMWH alone or low-dose aspirin (LDA) alone in women at high risk of PE.

### Search strategy

PubMed, Cochrane Library, Embase, and ClinicalTrials.gov databases were searched from their inception to April 2022. We also searched Embase, OVID MEDLINE and OVID MEDLINE in-process using the OVID platform. The search terms that we used in the Pubmed database were as follows: #1 “pre-eclampsia” [title/abstract] OR “preeclampsia” [title/abstract] OR “PE” [title/abstract] OR “eclampsia” [title/abstract]; #2 “heparin” [title/abstract] OR “Low-Molecular-Weightheparin” [title/abstract] OR “LMWH” [title/abstract]; #3 “aspirin” [title/abstract]; #4 #1 AND #2 AND #3. The databases were searched for published studies in English, including systematic reviews and meta-analysis, that were related to the treatment with LMWH or heparin (with LDA) in women at high risk of PE. After the search is complete, the documents were exported in full text format.

### Inclusion and exclusion criteria

Inclusion criteria were: (1) randomized controlled trials (RCTs) studies comparing LDA with LMWH or heparin for prevention of PE; (2) studies including women who had any known high risk factors for PE and its complications, such as adverse obstetric history of previous PE, small-for-gestational age (SGA) or FGR, placental abruption, and medical history including thrombophilia, autoimmune disease, and other chronic diseases; (3) published paper.

Animal experiments and non-RCTs were excluded from the meta-analysis. In addition, studies were excluded if they included other treatment options.

The primary outcomes were PE and live birth rate, with secondary outcomes including placental abruption, severe PE (sPE), gestational hypertension, FGR or SGA, fetal and neonatal death and HELLP (hemolysis, elevated liver enzymesand low platelet count) syndrome. We also analyzed the presence of adverse events such as intrapartum or postpartum hemorrhage.

In this context, it is recommended that women are diagnosed with PE if they present with the following severe features: systolic blood pressure of 140 mmHg or more or diastolic blood pressure of 90 mmHg or more on two occasions at least 4 h apart after 20weeks of gestation in a woman with a previously normal blood pressure plus proteinuria, defined asurinary excretion of 300 mg or more per 24 h urine collection (or this amount extrapolated from a timed collection)orc Protein/creatinine ratio of 0.3 mg/dL or more orc Dipstick reading of 2+(used only if other quantitativemethods not available) (14). The women are diagnosed with sPE when the following conditions are met: systolic blood pressure ≥160 mm Hg and/or diastolic blood pressure ≥110 mm Hg; Urine protein ic blood pressure more or the meria was strongly positive; brain nerve symptoms persist and worsen; persistent epigastric pain; serum aspartate aminotransferase significantly increased, creatinine >106 μmol/L; hypoalbuminemia, pleural or peritoneal effusion, heart failure, pulmonary edema, oligohydramnios, abnormal blood system indicators and other manifestations may occur at the same time. In addition, the main distinction between SGA and FGR is that a SGA fetus may be small but not at increased risk of adverse perinatal outcome, while a fetus with size above the 10th percentile may be FGR and at increased risk of adverse perinatal and long-term outcome (15).

### Data extraction

Two authors (BX and YY) independently screened and retrieved the relevant literatures. Then, after reading the title and abstract of the article, they excluded the studies that did not meet the inclusion criteria. Full texts of final selected articles and data extraction were performed independently by other 2 researchers (LZ and YW), and any discrepancies were resolved by discussion.

The extracted data included names of the first authors of selected articles, basic information on studies (research design, patient groups, interventions, doses of medications, number of patients, duration of treatment), characteristics of patients (gestational age, participant condition), and reported outcomes (PE, live birth rate, sPE, gestational hypertension, placental abruption, FGR or SGA, fetal and neonatal death, HELLP syndrome and intrapartum or postpartum hemorrhage).

### Study quality

The risk of bias of RCTs was assessed using the Cochrane Collaboration's tool (16) based on seven domains: random sequence generation, allocation concealment, blinding of participants and personnel, blinding of outcome assessment, incomplete outcome data, selective reporting, and other biases. Two reviewers (YY and BX) independently finished the quality assessments. In the case of a disagreement, a third reviewer (LZ) was involved in finally reaching a consensus through negotiation.

### Statistical analysis

RevMan 5.3 software provided by the Cochrane Collaboration Network was used for meta-analysis of the included studies. The software was also used to draw a forest map. For continuous variables, data have been presented as the mean difference (MD) with 95% confidence interval (CI) between the experimental and control groups. For dichotomous outcome data, the risk ratio (RR) or odds ratio (OR) with 95% CI was calculated. The chi-square test was used to assess heterogeneity between studies, and combined with *I*^2^ to quantitatively judge the extent of heterogeneity. The fixed-effects model was applied at *P* > *0.1* and *I*^2^ < *40%*, which showed that there was little heterogeneity between studies. Otherwise, the random-effects model was used for analysis (17).

### Publication bias

Begg's and Egger's tests of asymmetry were performed using the statistical software Stata 12.0 to assess potential publication bias. The tests were also used to identify outliers (18).

## Results

### Study characteristics

**Figure 1:**
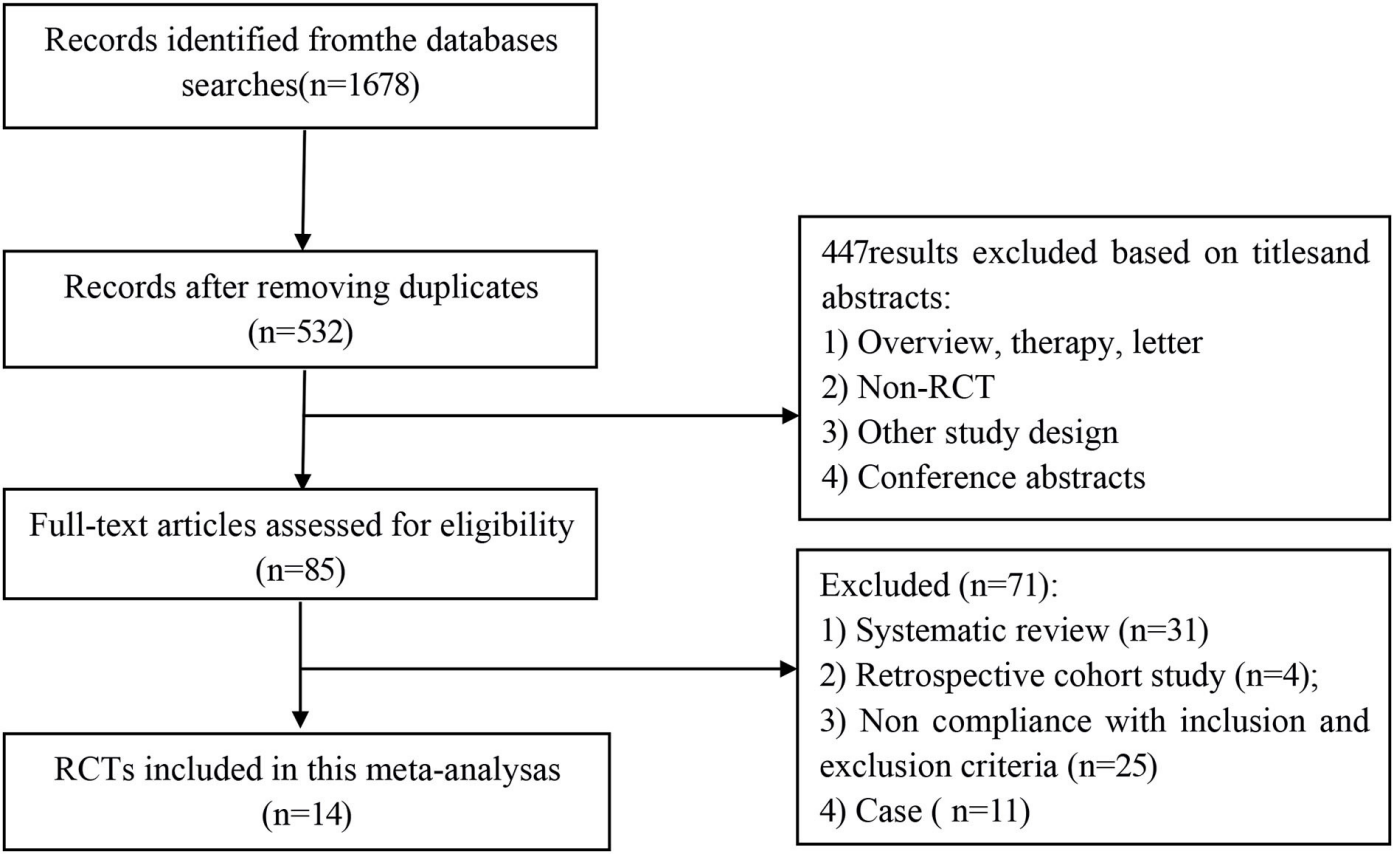
Flowchart of included studies.

Thousand six hundred and seventy eight articles were obtained from the search through the above mentioned databases. Of these, 1,146 articles were removed due to record duplication and 447 studies were excluded based on their titles and abstracts. Finally, a total of 14 studies that included 1,966 women were considered in the systematic review and meta-analysis based on the inclusion and exclusion criteria (Figure 1).

The baseline characteristics of the included studies were presented in the Table 1. Eight articles used enoxaparin (19, 20, 22, 24, 25, 27, 29, 30), three articles used dalteparin (21, 23, 26), one article used nadroparin (28), one did not specify the type of LMWH (32) and one used unfractionated heparin (31). The gestational age of included pregnant women varied among different studies, but all women were randomly divided between the first positive pregnancy test and 16 weeks' gestation. Because patients' conditions are heterogeneous across the different studies with different baseline characteristics, which may lead to heterogeneity in outcome measures, we performed subgroup analysis.

**Table 1:** Baseline characteristics of included studies in the meta-analysis.

**Study**	**Research design**	**Participant condition**	**No**.	**Interventions and dose**	**Duration of treatment (weeks)**
Karadag et al. (19)	A randomized controlled trial	Recurrent pregnancy loss patients with factor v leiden mutation	59	Enoxaparin 40 mg/d+asa 100 mg/d	Before termination of pregnancy
			61	ASA 100 mg/d	≤36weeks
Karadag et al. (19)	A randomized controlled trial	Recurrent pregnancy loss patients with factor v leiden mutation	59	Enoxaparin 40 mg/d+asa 100 mg/d	Before termination of pregnancy
			54	Enoxaparin 40 mg/d	≤36weeks
Groom et al. (20)	A multicenter open label randomized controlled trial	With a history of gestational hypertension, fgr, thrombophilia	72	Enoxaparin 4,000 iu/d+asa 100 mg/d	Before termination of pregnancy
			77	ASA 100 mg/d	≤36 weeks
Hoorn et al. (21)	A multicentre randomized controlled trial	With a history of gestational hypertension, sga	16	Dalteparin 5,000 iu/d+asa 80 mg/d	Before termination of pregnancy
			16	ASA 80 mg/d	≤36weeks
Haddad et al. (22)	A multicentre randomized controlled trial	With a history of spe	122	Enoxaparin 4,000 iu/d+asa 100 mg/d	Before termination of pregnancy
			122	ASA 100 mg/d	≤36 weeks
Carl et al. (23)	A open label randomized controlled trial	With a history of at least two consecutive abortions and thrombosis	45	Dalteparin 5,000 iu/d+asa 81 mg/d	Before termination of pregnancy
			43	ASA 81 mg/d	≤35weeks
Elmahashi et al. (24)	An open randomized clinical trial	A history ofthree or more consecutive miscarriages.	75	Enoxaparin 4,000 iu/day +asa 75 mg/d	34 weeks
			75	ASA 75 mg/d	
Gris et al. (25)	A monocentric, randomized controlled study	With a history of pe, fgr, placental abruption.	112	Enoxaparin 4,000 iu/d+asa 100 mg/d	Before termination of pregnancy
			112	ASA 100 mg/d	≤36 weeks
Vries et al. (26)	A multicentre randomized controlled trial	With a history of gestational hypertension, fgr, thrombophilia	70	Dalteparin 5,000 iu/d+asa 80 mg/d	Before termination of pregnancy
			69	ASA 80 mg/d	≤36weeks
Visser et al. (27)	A multicentre randomized controlled trial	With a history of recurrent abortion, but no thrombophilia	63	enoxaparin 40 mg/d+asa 100mg/d	Before termination of pregnancy
			76	ASA 100mg/d	≤35 weeks
Kaandorp et al. (28)	A multicentre randomized controlled trial	Had a history of unexplained recurrent miscarriage and were attemptingto conceive or were less than 6 weeks pregnant	123	nadroparin 2850 iu/d +asa 80 mg/d	Before termination of pregnancy
			120	ASA 80 mg/d	≤36weeks
Gris JC et al. (29)	A monocentric, randomized controlled study	Had a history of an unexplained pregnancy loss during the first intended pregnancy	80	Enoxaparin 4,000 iu/day +asa 100 mg/d	36 weeks
			80	ASA 100 mg/d	
Sergio et al. (30)	A monocentric, randomized controlled study	With a history of pe, fgr, but no thrombophilia	31	enoxaparin 4,000 iu/d+asa 100 mg/d	Before termination of pregnancy
			23	ASA 100 mg/d	≤36 weeks
Goel et al. (31)	A randomized controlled trial	With a history of two or more spontaneousabortions	33	heparin 5,000 iu/d+asa 80 mg/d	36 weeks
			39	ASA 80 mg/d	
Farquharson et al. (32)	A randomized controlled trial	With a history of at least three consecutive pregnancy lossesor two consecutive losses with proven fetal death after 10weeks' gestation	51	unspecified lmwh 5,000 iu/d+asa 75 mg/d	36 weeks
			47	ASA 75 mg/d	

### Risk of bias within studies

The Cochrane Collaboration's Risk of Bias tool (16) was used to evaluate the risk of bias of included studies. The results showed that the quality of most studies is acceptable. Most RCTs had an unclear risk of bias for allocation concealment, blinding of the outcome, and incomplete outcome data, considering that detailed information was not provided.

**Figure 2:**
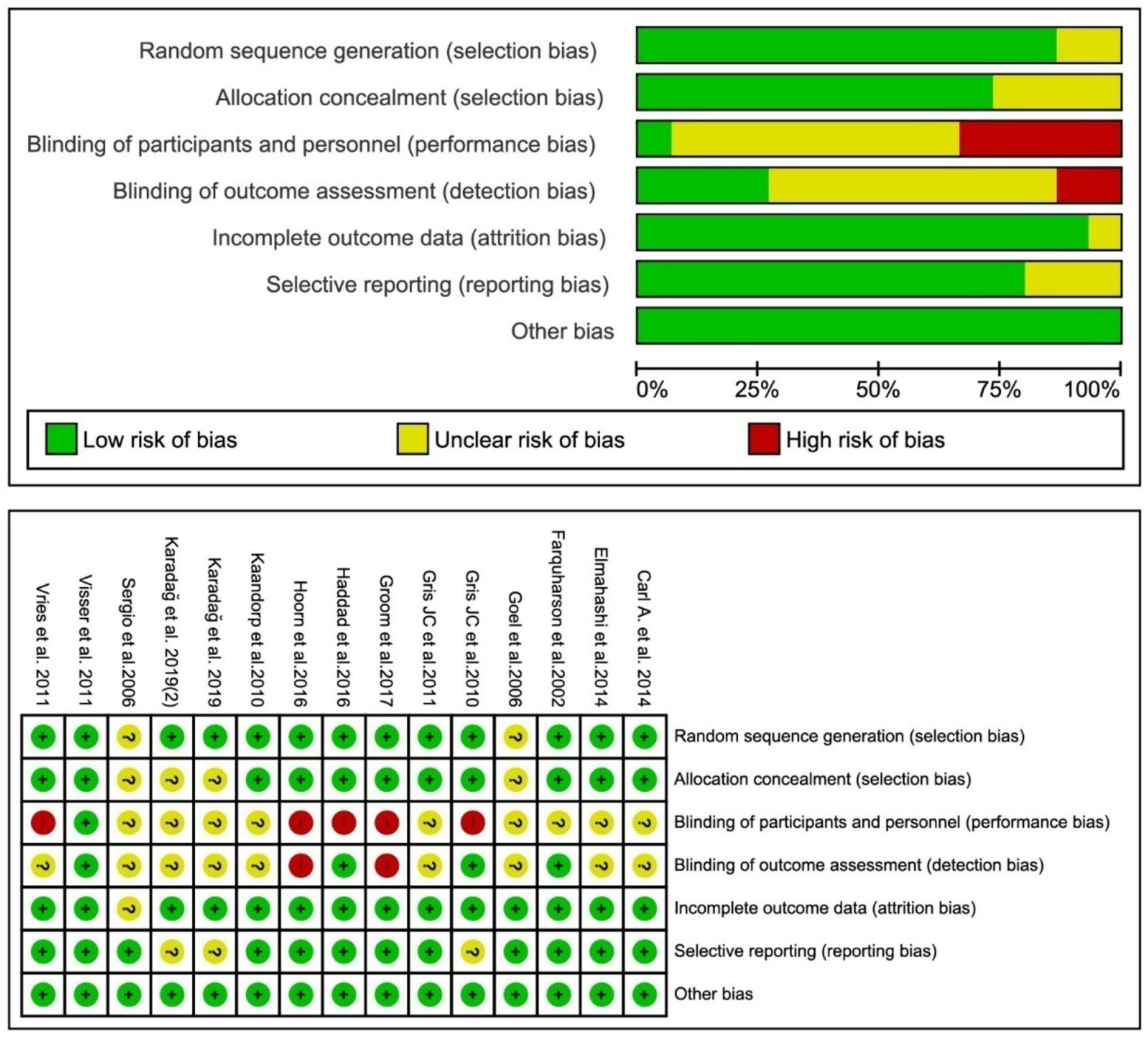
Risk of bias assessment of the studies.

The highest risk of bias occurred in the blinding of participants and personnel. This is because neither study personnel nor participants in five studies (20–22, 26, 29) were blinded to treatment assignment, as placebo injections were not considered to be ethically acceptable during pregnancy in. Quality assessment of the included studies is shown in Figure 2.

### Meta-analysis

Among all the included studies, six studies (20–22, 25, 26, 30) recruited women with a history of PE, two of which included only women with thrombophilia; eight studies (19, 23, 24, 27–29, 31, 32) recruited women with a history of miscarriages, one of them in women with thrombophilia and one of them including women with factor V Leiden mutation (FVL, a risk factor for deep vein thrombosis and pulmonary embolism). Due to the wide heterogeneity between the inclusion criteria of different studies, we performed subgroup analysis of the results according to the entry criteria.

### Primary outcomes

#### PE

**Figure 3:**
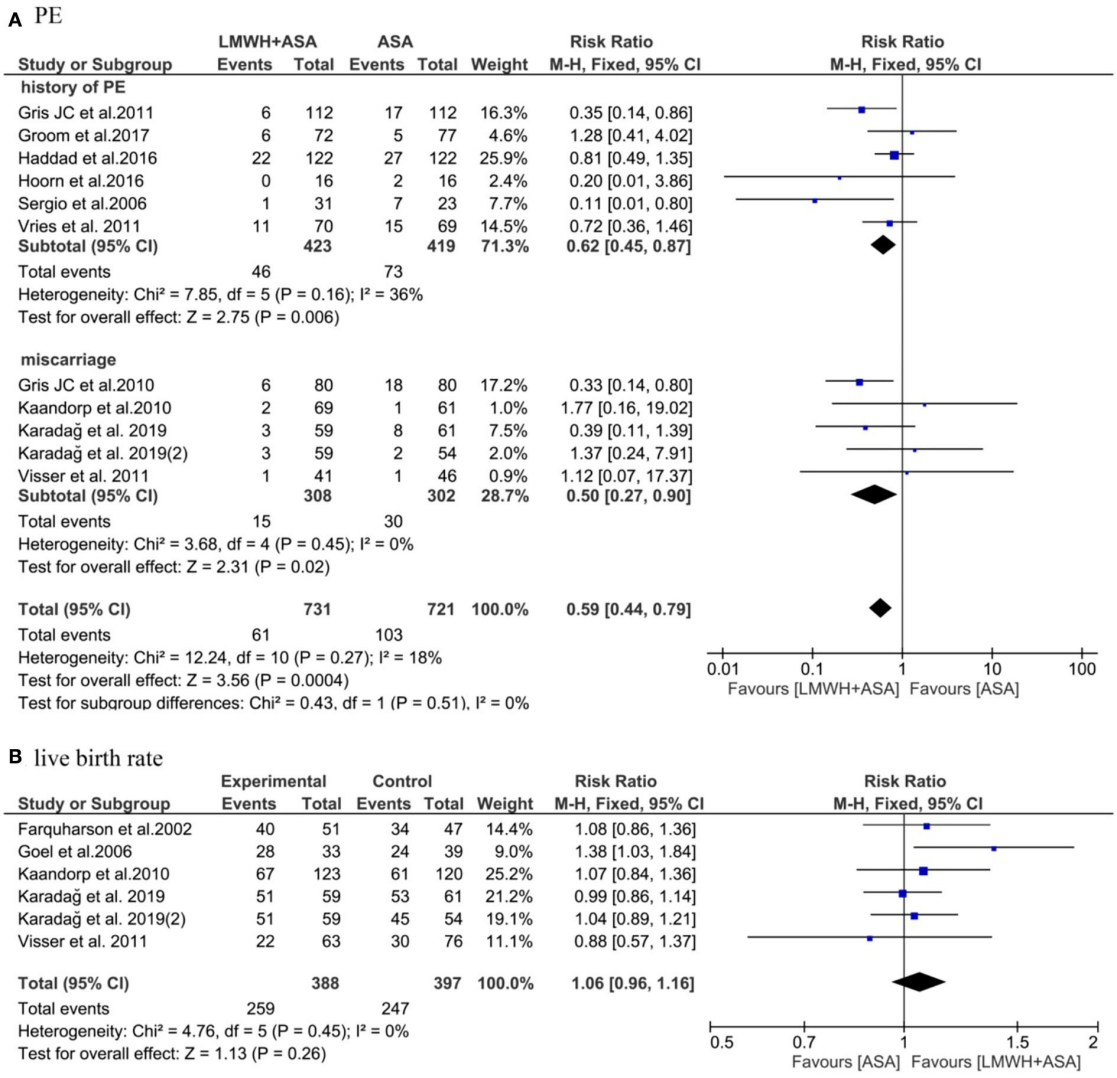
Meta-analysis of **(A)** PE and **(B)** live birth rate.

We observed that the data from ten studies (19–22, 25–30) included (*n* = 1,452), the pooled results obtained using a fixed-effects model showed that the addition of LMWH to LDA reduced the risk of PE (RR: 0.59, 95% CI: 0.44–0.79, *P* < *0.05*). In the subgroup analysis, the addition of LMWH to LDA reduced the risk of PE in women with a history of PE (RR: 0.62, 95% CI: 0.45–0.87, *P* < *0.05*) and miscarriages (RR: 0.50, 95% CI: 0.27–0.90, *P* < *0.05*), respectively (Figure 3A).

#### Live birth rate

Five studies (19, 27, 28, 31, 32) (n=785) reported the live birth rate, but these studies only included women with a history of miscarriages. The pooled results obtained using a fixed-effects model showed thatthe addition of LMWH to LDA did not improve the live birth rate (RR: 1.06, 95% CI: 0.96–1.16, *P* > 0.05) (Figure 3B).

### Secondary outcomes

#### Placental abruption

**Figure 4:**
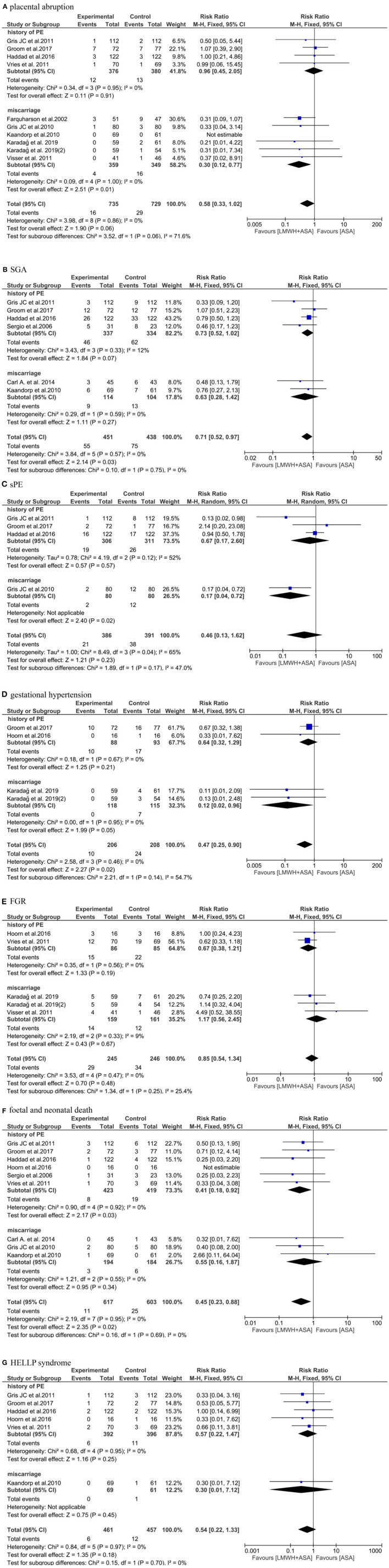
Meta-analysis of **(A)** placental abruption. **(B)** SGA. **(C)** sPE. **(D)** gestational hypertension. **(E)** FGR. **(F)** fetal and neonatal death. **(G)** HELLP syndrome.

We observed that the data from nine studies (19, 20, 22, 25–29, 32) included (*n* = 1,464), the pooled results obtained using a fixed-effects model showed that the addition of LMWH to LDA did not reduced the risk of placental abruption (RR: 0.58, 95% CI: 0.33–1.02, *P* = 0.06). In the subgroup analysis, the addition of LMWH to LDA did not reduced the risk of placental abruptionin women with a history of PE (RR: 0.96, 95% CI: 0.45–2.05, *P* = 0.91). Conversely, in women with a history of miscarriages, the addition of LMWH to LDA can reduce the risk of placental abruption (RR: 0.30, 95% CI: 0.12-0.77, *P* = 0.01) (Figure 4A).

#### SGA

Six studies (20, 22, 23, 25, 28, 30) (*n* = 889) reported the SGA, the pooled results obtained using a fixed-effects model showed that the addition of LMWH to LDA reduced the the risk of SGA (RR: 0.71, 95% CI: 0.52–0.97, *P* = *0.03*). But in the subgroup analysis, the addition of LMWH to LDA did not reduced the risk of SGA in women with a history of PE (RR: 0.73, 95% CI: 0.52–1.02, *P* = 0.07) and miscarriages (RR: 0.63, 95% CI: 0.28–1.42, *P* = 0.27), respectively (Figure 4B).

#### sPE

Four studies (20, 22, 25, 29) (*n* = 777) reported the sPE, the pooled results obtained using a random-effects model showed that the addition of LMWH to LDA did not reduced the the risk of sPE (RR: 0.46, 95% CI: 0.13–1.62, *P* = 0.23). In the subgroup analysis, the addition of LMWH to LDA did not reduced the risk of sPE in women with a history of PE (RR: 0.67, 95% CI: 0.17–2.60, *P* = 0.57). Conversely, in women with a history of miscarriages, the addition of LMWH to LDA can reduce the risk of sPE (RR: 0.17, 95% CI: 0.04–0.72, *P* = *0.02*) (Figure 4C).

#### Gestational hypertension

Three studies (19–21) (*n* = 414) reported the gestational hypertension, the pooled results obtained using a fixed-effects model showed that the addition of LMWH to LDA reduced the the risk of gestational hypertension (RR: 0.47, 95% CI: 0.25–0.90, *P* = 0.02). In the subgroup analysis, the addition of LMWH to LDA did not reduced the risk of gestational hypertension in women with a history of PE (RR: 0.64, 95% CI: 0.32–1.29, *P* = 0.21). However, in women with a history of miscarriages, the addition of LMWH to LDA can reduce the risk of gestational hypertension (RR: 0.12, 95% CI: 0.02–0.96, *P* = 0.05) (Figure 4D).

#### FGR

Four studies (19, 21, 26, 27) (*n* = 491) reported the FGR, the pooled results obtained using a fixed-effects model showed that the addition of LMWH to LDA did not reduced the the risk of FGR (RR: 0.85, 95% CI: 0.54–1.34, *P* = 0.48). In the subgroup analysis, the addition of LMWH to LDA also did not reduced the risk of FGR in women with a history of PE (RR: 0.67, 95% CI: 0.38–1.21, *P* = 0.19) and miscarriages (RR: 1.17, 95% CI: 0.56–2.45, *P* = 0.67), respectively (Figure 4E).

#### Fetal and neonatal death

Nine studies (20–23, 25, 26, 28–30) (*n* = 1,220) reported the fetal and neonatal death, the pooled results obtained using a fixed-effects model showed that the addition of LMWH to LDA reduced the risk of fetal and neonatal death (RR: 0.45, 95% CI: 0.23–0.88, *P* = 0.02). In the subgroup analysis, the addition of LMWH to LDA also can reduced the risk of fetal and neonatal deathin women with a history of PE (RR: 0.41, 95% CI: 0.18–0.92, *P* = 0.03), but the addition of LMWH to LDA did not reduce the risk of fetal and neonatal death in women with a history of miscarriages (RR: 0.55, 95% CI: 0.16–1.87, *P* = 0.34) (Figure 4F).

#### HELLP syndrome

Six studies (20–22, 25, 26, 28) (*n* = 918) reported the HELLP syndrome, the pooled results obtained using a fixed-effects model showed that the addition of LMWH to LDA did not obviously reduced the risk of HELLP syndrome (RR: 0.54, 95% CI: 0.22–1.33, *P* = 0.18). In the subgroup analysis, the addition of LMWH to LDA also did not reduced the risk of HELLP syndrome in women with a history of PE (RR: 0.57, 95% CI: 0.22–1.47, *P* = 0.25) and miscarriages (RR: 0.30, 95% CI: 0.01–7.12, *P* = 0.45), respectively. Although the results were not statistically different, it can be seen from the Figure 4G that LDA combined with LMWH is more beneficial to prevent the occurrence of HELLP syndrome and is a more favorable treatment option (Figure 4G).

### Safety outcomes

#### Intrapartum or postpartum hemorrhage

**Figure 5:**
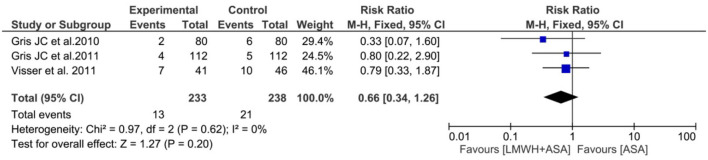
Meta-analysis of intrapartum or postpartum hemorrhage.

Three studies (25, 27, 29) (*n* = 471) reported the intrapartum or postpartum hemorrhage, the pooled results obtained using a fixed-effects model showed that the addition of LMWH to LDA did not increased the risk of intrapartum or postpartum hemorrhage (RR: 0.66, 95% CI: 0.34–1.26, *P* = 0.20) (Figure 5).

### Publication bias

The results of Begg's and Egger's tests showed no evidence of publication bias for all the outcomes except PE, as all the results showed *P* > 0.05 (Table 2). However, there was evidence of publication bias for PE, as the *P*-value from Egger's tests was 0.007, the P value from Begg's tests was 0.02.

**Table 2:** Results of Egger's and Begg's tests.

**Test**	**Begg's**	**Egger's**
	***P-*** **value**	
PE	0.02	0.007
live birth rate	0.13	0.30
placental abruption	0.18	0.06
SGA	0.45	0.67
sPE	0.73	0.51
gestational hypertension	1.00	0.98
FGR	0.46	0.58
fetal and neonatal death	0.27	0.13
HELLP syndrome	0.45	0.14
intrapartum or postpartum hemorrhage	1.00	0.70

## Discussion

The results of this systematic review and meta-analysas showed that among women with a history of PE, LMWH combined with LDA can effectively reduce the incidence of PE and fetal and neonatal death. However, in the prevention of placental abruption, sPE, gestational hypertension, SGA and FGR, compared with LDA or LMWH alone, the combination of the drugs appeared to have no benefit. Nevertheless, the combination of drugs reduced the incidence of gestational hypertension, sPE, SGA and FGR in women who had previously developed PE. In women with a history of miscarriage, LMWH combined with LDA can significantly reduce the incidence of PE, placental abruption, sPE and gestational hypertension. However, there was no significant difference between the combined use of the two drugs and the use of a single drug in improving the live birth rate of fetus and reducing the incidence of SGA, FRG and fetal and neonatal death. Despite the results, the combination of drugs increased the number of newborns and decreased the number of SGA and fetal and neonatal death in women with previous miscarriages. In addition, no major side effects such as intrapartum or postpartum hemorrhage were observed in women treated with LMWH combined with LDA, and the combined medication reduced the incidence of HELLP syndrome.

During our search in the above mentioned databases, we found a meta-analysis (8) reported on LMWH combined with aspirin in preventing PE and SGA. The meta-analysis involved 8 RCTs, which were divided into two subgroups according to women's history of PE or miscarriage, which is roughly the same as our study grouping criteria. The results of this study showed that the combination of drugs could not effectively reduce the incidence of PE and SGA in women with a history of miscarriage. However, after we increased the sample size, we came to a conclusion inconsistent with Roberge et al. (8). We found that in high-risk women, combination therapy not only reduced PE, but also reduced fetal and neonatal death.

Aspirin inhibits the production of prostaglandin and thromboxane A2 by irreversible acetylation of serine residues in the active center of cyclooxygenase 1 (COX-1) and cyclooxygenase 2 (COX-2), and finally inhibits platelet aggregation (33), thereby reducing thrombosis. Aspirin can also inhibit the production of thromboxane and prostacyclin in trophoblast, thus effectively preventing the contraction of placental blood vessels and avoiding insufficient placental blood flow. Additionally, aspirin plays a therapeutic role in preeclampsia through anti platelet aggregation, prevention of oxidative stress, improvement of cell apoptosis, etc (34, 35).

LMWH strengthens its inactivation of coagulation factors IIa, IXa and Xa by combining with antithrombin, thus reducing the generation of corresponding coagulation factors. It not only has the effects of anticoagulant, preventing placental thrombosis and placental infarction (36), but also has the effects of anti-inflammatory, anti-tumor and promoting angiogenesis (10, 37). LMWH can promote the conversion of plasminogen to plasmin, reduce blood viscosity, change blood rheology, inhibit vasoconstriction, improve organ blood perfusion, protect vascular endothelium, strengthen placental exchange function, increase fetal urine volume and amniotic fluid volume, promote fetal growth and development, and improve perinatal prognosis (10). With further research, it was found that LMWH not only acts as an anticoagulant, but also has an obvious effect on the development and invasion of trophoblast (38). These results make LMWH as a potential drug for the prevention of PE and SGA.

Studies have shown that LDA combined with LMWH may offset the adverse reactions caused by some drugs and increase the effect of the two drugs (39). For pregnant women with underlying diseases, LDA should not be given only. For the impact of factors such as hypercoagulability and thrombophilia on placenta and fetus, anticoagulant drugs such as LMWH may also be needed (40). Our results also show that LMWH combined with LDA can significantly improve the pregnancy outcome of high-risk women without increasing the risk of bleeding.

The strengths of our study include the inclusion of more RCT studies and more patients comparing the effects of combination and single drug than other meta-analysis. Although there may be heterogeneity in our results, it also proves to some extent that our results are applicable to women with high risk factors for PE and its complications. At the same time, we also evaluated the bleeding risk of combined drugs, which is also a key safety index to be considered in the process of clinical use.

This study has some limitations. First, the quality grades of the included studies were inconsistent. Among them, five studies (20–22, 26, 29) did not adopt blind design, and the random allocation method of two studies (30, 31) was unclear. Second, because many women chose to withdraw from the trial study, there was uncertainty about the treatment compliance of the subjects. Third, four of all included RCTs in recurrent miscarriage did not report whether LMWH combined with LDA could reduce the incidence of PE. In addition, only three of the 14 included studies reported on intrapartum or postpartum hemorrhage, leading to publication bias in the results. Fourth, the study did not evaluate the impact of long-term maternal and infant outcomes. More large sample, multicenter and long-term follow-up studies are needed to confirm the long-term impact of combined drugs on maternal and infant outcomes. Finally, the Begg's and Egger's test results of PE showed potential publication bias, indicating that the statistical analysis results may be unstable, and it is recommended to carry out studies with higher quality and more rigorous experimental design.

Therefore, the efficacy of aspirin combined with LMWH is better than that of single drug, and can improve maternal and infant outcomes to a certain extent. It is worthy of clinical promotion, but it needs to be further verified by strictly designed, large sample and multi center randomized controlled trials.

## Conclusion

The results of this meta-analysis showed that LMWH combined with LDA can effectively improve the pregnancy outcome of women with high risk factors and reduce the incidence of PE, SGA, gestational hypertension, fetal and neonatal death. At the same time, there are three studies that report women's bleeding. The results show that the combination of drugs does not increase the risk of bleeding, but such results lack the support of large sample size studies. The clinical safety analysis of LMWH combined with LDA in patients with PE should be more carried out.

## Data availability statement

The original contributions presented in the study are included in the article/supplementary material, further inquiries can be directed to the corresponding authors.

## Author contributions

LZ and YaW planned and designed the study. LZ, BX, YY, and YuW screened studies and performed the systematic review and extracted data from included studies. YY, BX, and LZ analyzed the data and performed the quality assessment of studies. LZ drafted the manuscript. All authors contributed to the discussion section of the manuscript. All authors contributed to the article and approved the submitted version.
